# An evaluation of the efficacy of percutaneous reduction and screw fixation without bone grafting in Sanders Type-II and Type-III displaced intra-articular calcaneal fractures

**DOI:** 10.1186/s12891-022-05515-2

**Published:** 2022-06-10

**Authors:** Gang Luo, Chongyin Fan, Peili Gao, Wei Huang, Weidong Ni

**Affiliations:** Department of Orthopaedic Surgery, The First Affiliated Hospital of Chongqing Medical University, 1 Youyi Rd, Chongqing, 400016 China

**Keywords:** Calcaneum, Intra-articular fractures, Bone grafting, Minimally invasive, Percutaneous fixation

## Abstract

**Background:**

The aim of this retrospective monocentric study was to investigate the clinical efficacy of percutaneous reduction and screw fixation without bone grafting in Sanders Type-II and Type-III displaced intra-articular calcaneal fractures (DIACFs).

**Methods:**

The medical records of calcaneal fractures patients who were admitted to our department from January 2018 to January 2020 were retrospectively reviewed, and those meeting the inclusion criteria were fnally included for analysis. All patients were treated with percutaneous reduction and screw fixation, and no patients received bone grafting. The radiologic parameters evaluated included the BÖhler angle and the calcaneal height. In addition, the American Orthopaedic Foot and Ankle Society (AOFAS) hindfoot scores, Maryland Foot Score (MFS), and visual analog scale (VAS) score were determined.

**Results:**

Thirty-eight patients with Sanders Type-II and Type-III DIACFs were finally included, including 30 males and 8 females aged 21 to 61 years [(42.6 ± 9.6) years]. According to the Essex-Lopresti classification, 27 of the fractures were the tongue type, and 11 were the joint compression type. According to the Sanders classification, 27 of the fractures were type II, and 11 were type III. Immediately postoperatively, the calcaneal height had recovered to 39.8 ± 2.1 mm, the BÖhler angle had recovered from 4.2° ± 13.6° preoperatively to 27.2° ± 3.4° (*P* = 0.000). All patients were followed up for 18–42 months [(25.2 ± 9.5) months]. All fractures healed. No differences were found in the outcome measures six-months postoperatively (BÖhler angle, *p* = 0.24; calcaneal height, *p* = 0.82) or at final follow-up (BÖhler angle, *p* = 0.33; calcaneal height, *p* = 0.28) compared to the immediately postoperative values. At the final follow-up, the AOFAS score was 91.7 ± 7.4 points, with an excellent and good rate of 92.1%; the MFS was 90.3 ± 7.8 points, with an excellent and good rate of 92.1%; and the VAS score was 2.2 ± 1.5 points. None of the patients had incision complications, and one patient developed traumatic arthritis.

**Conclusion:**

Percutaneous reduction and screw fixation without bone grafting in Sanders Type-II and Type-III DIACFs can achieve good recovery and maintenance of the BÖhler angle and calcaneal height. Moreover, it has the advantage of a low complication rate.

## Background

Displaced intra-articular calcaneal fractures (DIACFs) are a common type of calcaneal fracture, and functional status assessment (SF-36 score) of DIACF patients showed that their functional prognosis is far worse than that of patients with other fractures [1]. The pendulum for treating these injuries has swung between nonoperative and surgical management, with the most recent shift being towards operative restoration of calcaneal height and anatomic reduction of the subtalar articular surface [2–7]. Open reduction and internal fixation (ORIF) through an extended lateral approach has been the most frequently utilised technique for surgically restoring the calcaneal height and BÖhler angle over the last three decades [8–11].

Nevertheless, whether bone grafting is needed in the treatment of DIACFs is still controversial. Proponents believe that using bone-graft or substitute can fill the cavity, enhance the mechanical strength, promote fracture healing, and provide better maintenance of calcaneal height and BÖhler angle, which can help prevent later joint surface collapse and the occurrence of posttraumatic arthritis [12–15]. However, the recent meta-analysis [16] shows that the use of bone grafting for the management of calcaneal fractures requires additional substantiation. Those who suggest no bone grafting think that the regenerative capacity of the highly vascular cancellous bone of the calcaneus is enough to fill the void over time [8, 17], and there is no need for augmentation if adequately stable internal fixation is achieved [18–21]. In the treatment of DIACFs, the ability to attain an effective fixation construct is an important consideration for bone grafting.

Many studies [22–25] have confirmed that minimally invasive treatment of DIACFs with percutaneous reduction and screw fixation can achieve the same clinical efficacy as ORIF and significantly reduce the complications related to wound healing. Therefore, minimally invasive percutaneous treatment of DIACFs has become popular in recent years. However, there is little literature on whether bone grafting is needed in the treatment of DIACFs with this technique. We believe that minimally invasive percutaneous treatment of DIACFs can greatly protect the blood supply of the fracture area, prevent further bone loss caused by reduction during ORIF, and facilitate fracture healing. As long as effective strength fixation can be achieved, bone grafting is not necessary to further support the articular surface. Therefore, in the present study, we explore the results of minimally invasive percutaneous treatment of Sanders Type II and Type III DIACFs with no bone grafting. The present study provides important information on whether bone grafting is needed in minimally invasive percutaneous treatment of Sanders Type-II and Type-III DIACFs.

## Methods

### Subjects

The medical records of calcaneal fractures patients who were admitted to our department from January 2018 to January 2020 were retrospectively analyzed.

The inclusion criteria were as follows: 1) age ≥ 16 years; 2) unilateral Sanders Type-II or Type-III DIACFs; 3) fresh fracture (within 3 weeks); 4) closed fracture;5) the operative method was percutaneous reduction and screw fixation without bone grafting; and 6) the follow-up data was complete.

The exclusion criteria were as follows: 1) severe osteoporosis; 2) multiple injury; 3) severe medical diseases (severe renal insufficiency, hyperthyroidism, stroke or myocardial infarction in nearly 3 months); and 4) inability to tolerate surgery.

The study was approved by the Medical Ethics Committee of the First Affiliated Hospital of Chongqing Medical University (Chongqing, China)(Ethical NO. 2020–414), and was performed in accordance with the ethical standards of the Declaration of Helsinki of 1964. All patients signed an informed consent form.

### Preoperative management

Lateral, anteroposterior, and axial X-rays, CT and 2-dimensional (2D) reconstruction of the calcaneus were performed to assess the morphological changes of the calcaneus and the degree of articular surface collapse and to inform Sanders and Essex-Lopresti classification. After injury, the limb was elevated, and an ice pack was applied to decrease the swelling. There was no need to wait for skin "wrinkle signs" before surgery.

### Surgical techniques

**Fig. 1:**
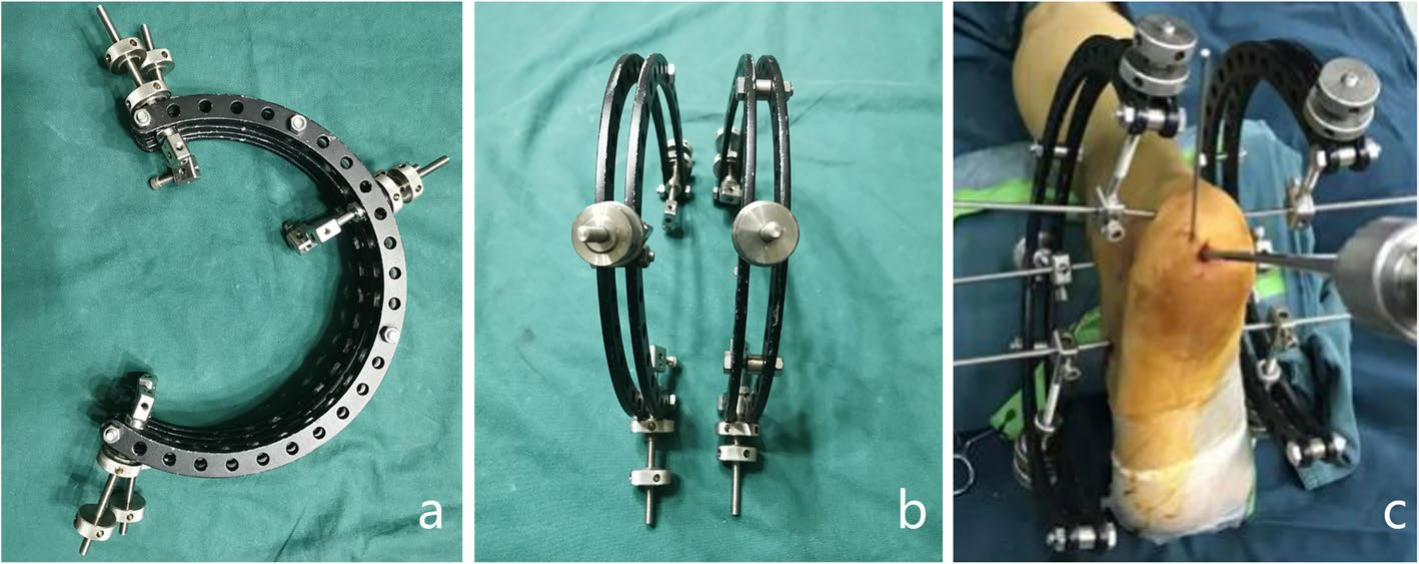
Homemade TCDR (Chinese Patent No.: 20192254154.4) (**a,b**). Clinical photograph showed homemade TCDR was set up on both side of the calcaneal (**c**)

**Fig. 2:**
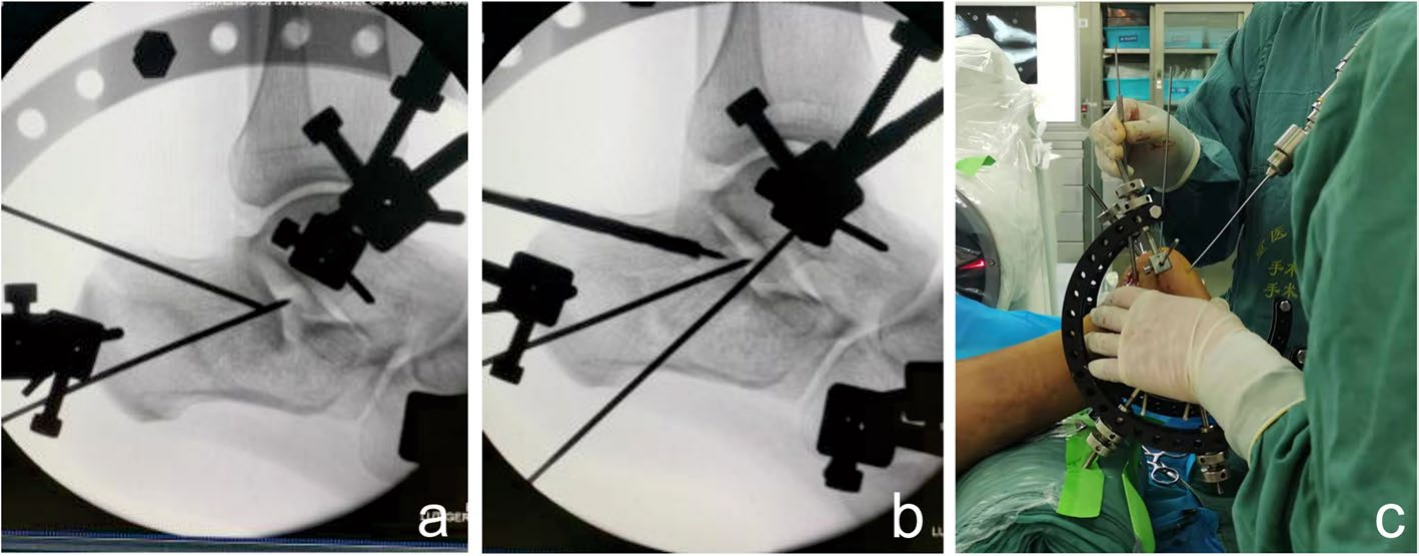
Essex-Lopresti manoeuvre technique. A 2.0 mm K-wire was drilled into tongue-type fragment under fluoroscopic guidance (**a**), then a hollow reduction tool was inserted along the k-wire to reduce the tongue-type fragment (**b**).Clinical photograph showing reduction of the tongue-type fragment with use of a hollow reduction tool (**c**)

**Fig. 3:**
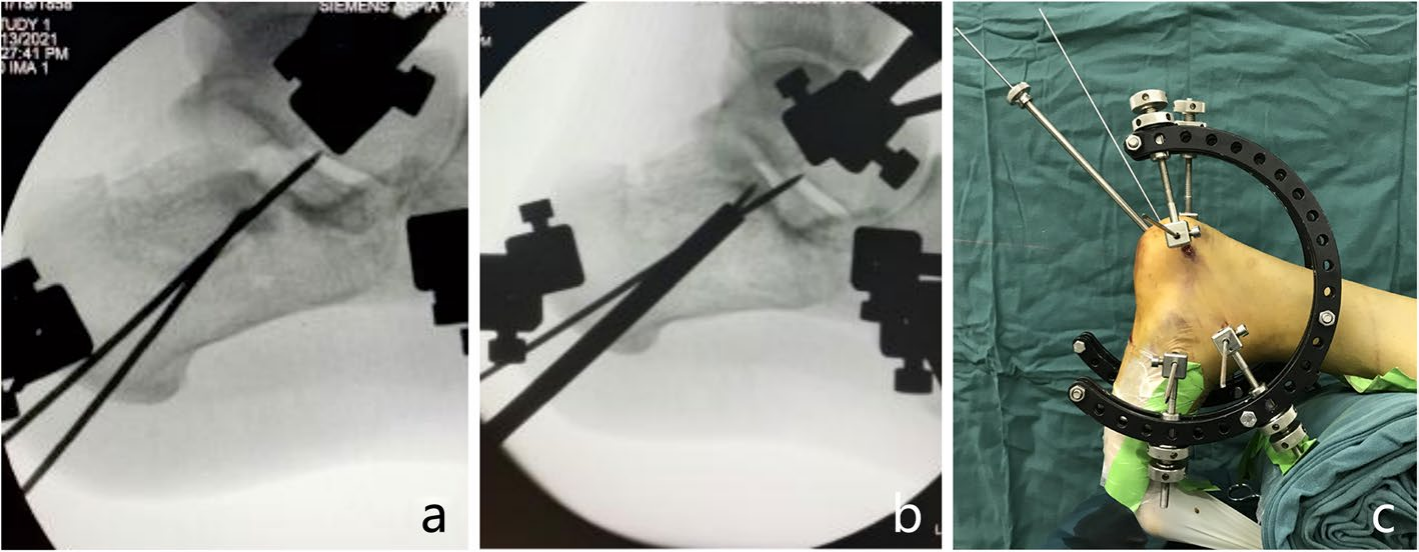
Percutaneous prying and jacking reduction technique. A 2.0 mm K-wire was drilled into joint depression-type fragment under fluoroscopic guidance (**a**), then a hollow punch was inserted along the k-wire to reduce the joint depression-type fragment (**b**). Clinical photograph showing reduction of the depression-type fragment with use of a hollow punch (**c**)

**Fig. 4:**
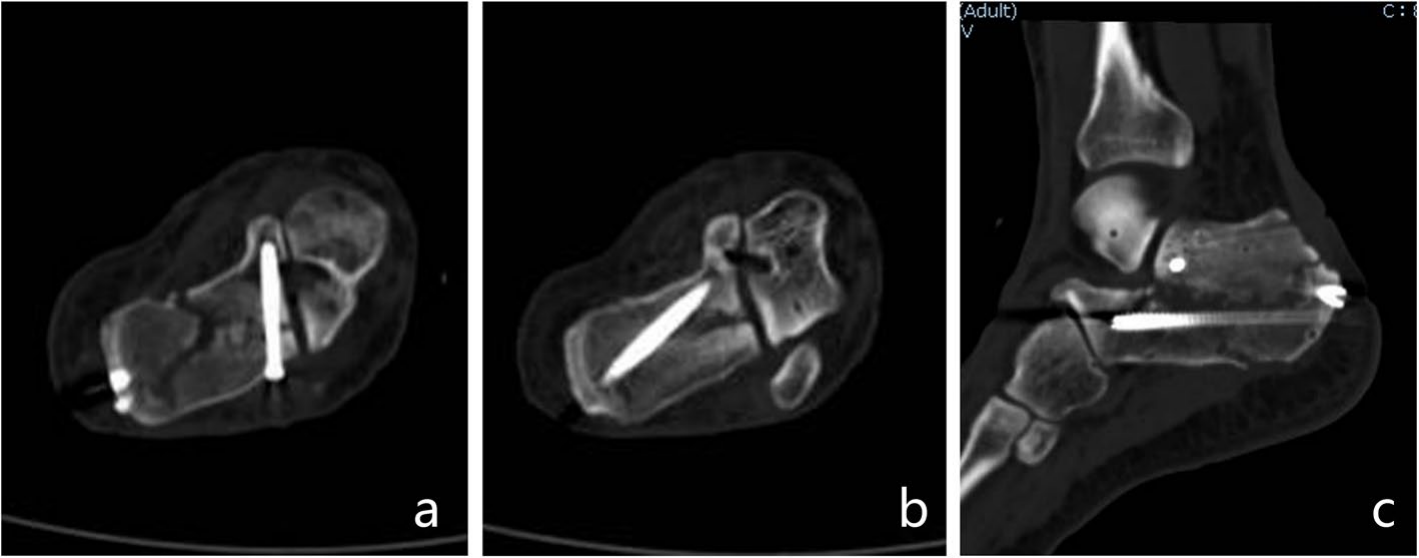
The specific position of the screw.The first screw was from calcaneal colliculus to sustentaculum tali (**a**), the second screw was from calcaneal tuberosity to sustentaculum tali (**b**),the third screw was from calcaneus tuberosity to calcaneus anterior tubercle (**c**)

The patient was placed in the prone position and received spinal or general anaesthesia with routine antibiotic prophylaxis prior to the operation. A 15 cm wooden pad was placed in front of the ankle joint. Three 3.0 mm K-wires were drilled into the calcaneal tuberosity, talar neck and cuboid bone. Then, a homemade tri-plane calcaneal distraction reductor (TCDR) (Chinese Patent No. 201922254154.4) was set up on both the medial and lateral sides of the calcaneus (Fig. 1). The tension between the three K-wires were adjusted with the TCDR to restore the height and length of the calcaneus, and the heel varus/valgus angulation. These imaging parameters were confirmed using C-arm fluoroscopy in calcaneal lateral and axial views. Then, the medial calcaneus column was fixed with 1–2 Kirschner wires temporarily. According to the Sanders and Essex-Lopresti classification, the Essex-Lopresti manoeuvre (Fig. 2) or percutaneous prying and jacking reduction technique (Fig. 3) were used to reduce the articular surface fracture fragments. After the articular surface fracture fragments were reduced, the lateral wall of the calcaneum was pressed by hand to restore the calcaneus width by reducing the bulging of the lateral wall. After confirmation with C-arm fluoroscopy, one or two 3.5 mm or 4.3 mm cannulated screws were inserted through the calcaneal colliculus to the sustentaculum tali (Fig. 4a). For patients with large tongue fracture masses, an additional 3.5 mm or 4.3 mm cannulated screw was inserted from the tail end of the tongue fracture block to the plantar side of the foot. Finally, a 5.5 mm all-cortical cannulated screw was inserted along the calcaneal axis from the medial side of the calcaneal tuberosity to the sustentaculum tali to maintain the height of the calcaneum (Fig. 4b); another 5.5 mm all-cortical cannulated screw was inserted along the calcaneal long axis from the lateral side of the calcaneal tuberosity to the calcaneal anterior tubercle to maintain the calcaneum length (Fig. 4c). The wound was then closed layer by layer without drain insertion.

### Postoperative management

Functional training was started immediately postoperatively, including active and passive flexion and extension of the toes and ankles. Inversion and eversion exercises (including ankle and subtalar joints) were started 2 weeks postoperatively, and circle exercises were started 1 month postoperatively. The weight-bearing time was determined according to the healing condition of the fracture. Generally, 8–12 weeks postoperatively, complete weight-bearing was started, as were supported squat exercises. Outpatient reexamination was generally carried out 1, 3, 6 and 12 months postoperatively, and follow-up was performed once a year after 1 year postoperatively.

### Clinical evaluation

Functional outcomes were assessed by the American Orthopaedic Foot and Ankle Society (AOFAS) hindfoot scores and Maryland Foot Score (MFS). The AOFAS.

ankle-hindfoot scale consists of subjective and objective variables classified into three major categories: pain, function, and alignment. The MFS has the same major categories. The total scores for both scales range from 0 to 100, with lower scores indicating greater impairment. For this evaluation, the AOFAS scores were divided into the same categories as those used for the MFS: a score of 90 to 100 was graded as excellent; 75 to 89, as good; 50 to 74, as fair; and less than 50 points, as poor. Pain was assessed by visual analog scale (VAS) with a range of 0 to 10, with 0 indicating the best possible result and 10 indicating the worst possible result. Postoperative wound-related complications included incisions and other related complications. Traumatic arthritis was comprehensively evaluated by its clinical symptoms and imaging manifestations.

### Radiological evaluation

Lateral and axial radiographs and CT scans were obtained immediately postoperatively to assess the reduction. Lateral and axial radiographs of the injured foot were performed at each follow-up evaluation to assess loss of reduction. The calcaneal anatomical parameters, including the Böhler angle and calcaneal height, were measured by radiographs six months postoperatively and at the final follow-up.

### Statistical analysis

IBM SPSS Statistics 26 software (IBM Corp., Armonk, NY) was used to analyse the normality of the distribution of the measurement data (height of calcaneum, Böhler angle, AOFAS score, MFS, VAS). Those with a normal distribution are represented as x ± s. A paired sample t test was used to compare different time points. *P* < 0.05 indicated a statistically significant difference.

## Results

**Fig. 5:**
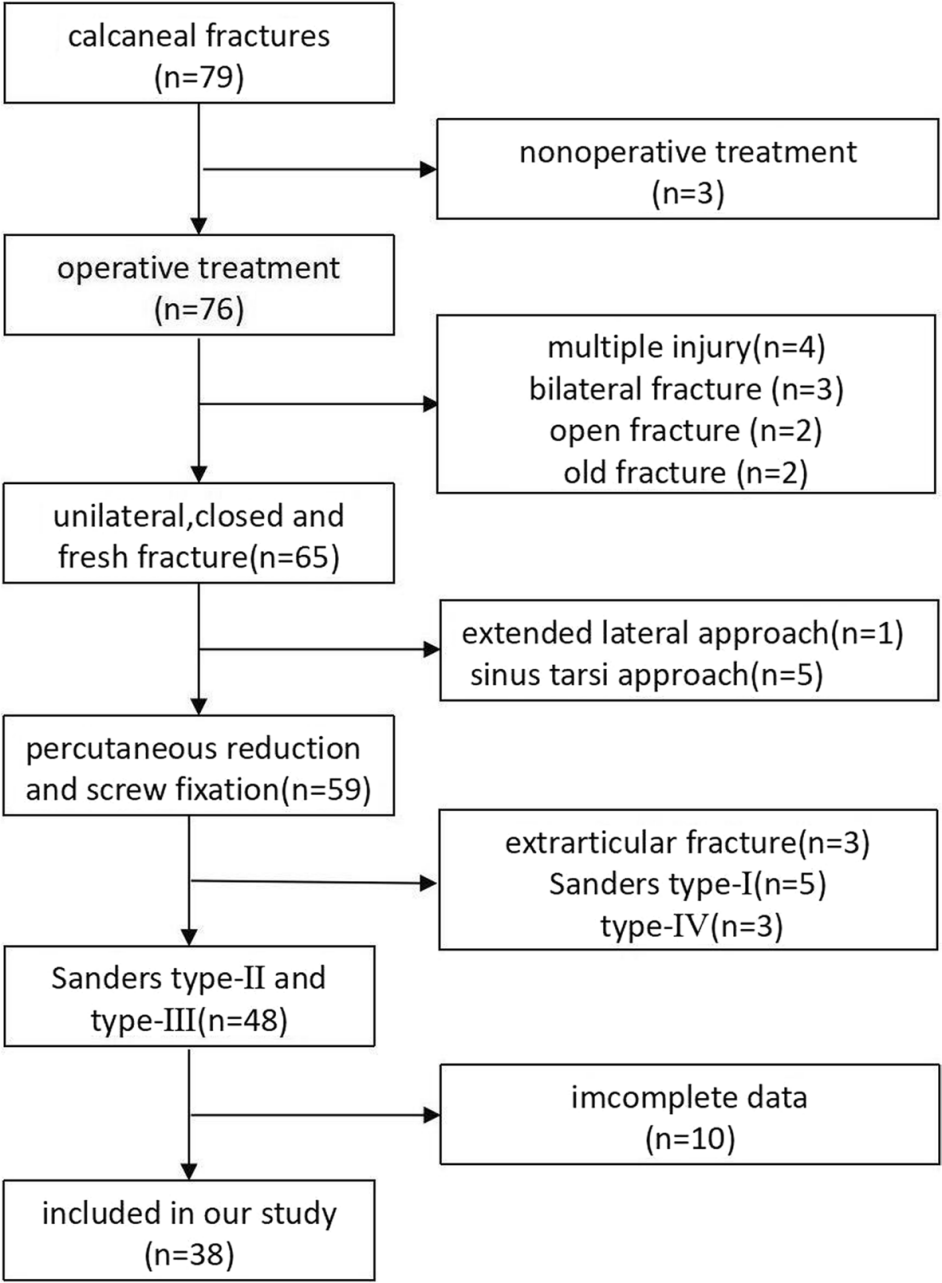
Patient selection flow chart

A total of 38 patients were finally included in this study (Fig. 5), including 30 males and 8 females aged 21 to 61 years [(42.6 ± 9.6) years]. According to the Essex-Lopresti classification, 27 were the tongue type, and 11 were the joint compression type. According to the Sanders classification, 27 were type II, 11 were type III. Thirty-one were fall injuries, and 7 were traffic injuries. The preoperative waiting time was 3.6 ± 0.9 d, the operation time was 88.4 ± 9.0 min, and the hospital stay was 7.2 ± 1.0 d (Table 1).

**Table 1:** Patient demographics

		all	male	female
Sex (M/F)		38	30	8
Age (years), (x ± s)		42.6 ± 9.6	40.9 ± 9.4	48.9 ± 7.5
Side of injured (R/L)		26/12	21/9	5/3
Injure mechanism	Falling accident	31	24	7
	Traffic accident	7	6	1
Sanders classification	Type II	27	22	5
	Type III	11	8	3
Essex-Lopresti classification	Tongue-type	27	23	4
	Joint depression-type	11	7	4
Preoperative waiting time		3.6 ± 0.9	3.5 ± 0.8	3.8 ± 1.0
operation time		88.4 ± 9.0	86.4 ± 8.0	95.8 ± 8.8
Hospital stay		7.2 ± 1.0	7.2 ± 1.0	7.3 ± 1.0

All patients were followed up for 18–42 months [(25.2 ± 9.5) months]. All fractures healed. All of the patients could wear shoes properly. One patient chose to retire due to his approaching retirement age after surgery, while the rest returned to their working positions or living conditions before injury. AOFAS scores averaged 88.2 ± 7.1 points six months postoperatively and 91.7 ± 7.4 points at the final follow-up; 21 cases were excellent, 14 cases were good, and 3 cases were fair, with an excellent/good rate of 92.1%. The MFS averaged 86.7 ± 7.2 points six months postoperatively and 90.3 ± 7.8 points at the final follow-up; 19 cases were excellent, 16 cases were good, and 3 cases were fair. The excellent and good rate was 92.1%. The VAS score was 2.4 ± 1.6 points six months postoperatively and 2.2 ± 1.5 points at the final follow-up (Table 2).

**Table 2:** The function parameters at different follow-up time piont

		All	Sanders		Essex-Lopresti	
			Type II	Type III	Tongue -type	Joint depression-type
Six-months post-operative	AOFAS	88.2 ± 7.1	88.1 ± 3.5	84.7 ± 10.8	89.2 ± 5.7	85.7 ± 9.1
	MFS	86.7 ± 7.2	86.3 ± 3.3	83.0 ± 10.9	87.8 ± 6.0	84.1 ± 8.9
	VAS	2.4 ± 1.6	2.3 ± 1.5	2.8 ± 1.8	2.2 ± 1.5	3.1 ± 1.7
Final follow-up	AOFAS	91.7 ± 7.4	91.8 ± 3.5	88.6 ± 11.3	92.3 ± 6.4	90.3 ± 9.2
	MFS	90.3 ± 7.8	90.3 ± 3.3	87.0 ± 11.9	91.2 ± 6.6	88.1 ± 9.7
	VAS	2.2 ± 1.5	1.9 ± 1.3	2.7 ± 1.8	1.9 ± 1.4	2.7 ± 1.5

Immediately postoperatively, the calcaneal height had recovered to 39.8 ± 2.1 mm(Table 3), the BÖhler angle had recovered from 4.2° ± 13.6° preoperatively to 27.2° ± 3.4° (*P* = 0.000) (Table 4). No differences were found in the outcome measures six months postoperatively (BÖhler angle, *p* = 0.24; calcaneal height, *p* = 0.82) or at the final follow-up (BÖhler angle, *p* = 0.33; calcaneal height, *p* = 0.28) compared with immediately preoperatively (Table 3).

**Table 3:** The radiographic parameters between follow-up period and Immediate post-operative

	Follow-up period		Immediate post-operative	t	*p* Value
calcaneal height(mm)	Six-months post-operative	39.8 ± 2.0	39.8 ± 2.1	-0.23	0.82
	Final follow-up	39.9 ± 2.0		-1.10	0.28
BÖhler angle(°)	Six-months post-operative	27.0 ± 3.3	27.2 ± 3.4	1.20	0.24
	Final follow-up	27.0 ± 3.3		0.98	0.33

**Table 4:** Pre-operative and immediately post-operative BÖhler angle(°)

	Pre-operative	Immediate post-operative	t	*p* Value
Sanders II	1.9 ± 15.4	27.3 ± 3.2	-8.0	0.00
Sanders III	9.9 ± 3.1	27.1 ± 3.9	-14.2	0.00
Tongue-type	1.9 ± 15.3	26.9 ± 3.5	-7.9	0.00
Joint depression-type	9.8 ± 5.0	27.9 ± 3.0	-11.5	0.00
all	4.2 ± 13.6	27.2 ± 3.4	-9.8	0.00

None of the patients had complications related to wound healing. One patient developed traumatic arthritis approximately 1 year postoperatively, which manifested as pain in the tarsal sinus area when walking on an uneven road surface. However, oral nonsteroidal anti-inflammatory painkillers significantly improved the symptoms without surgical treatment.

**Fig. 6:**
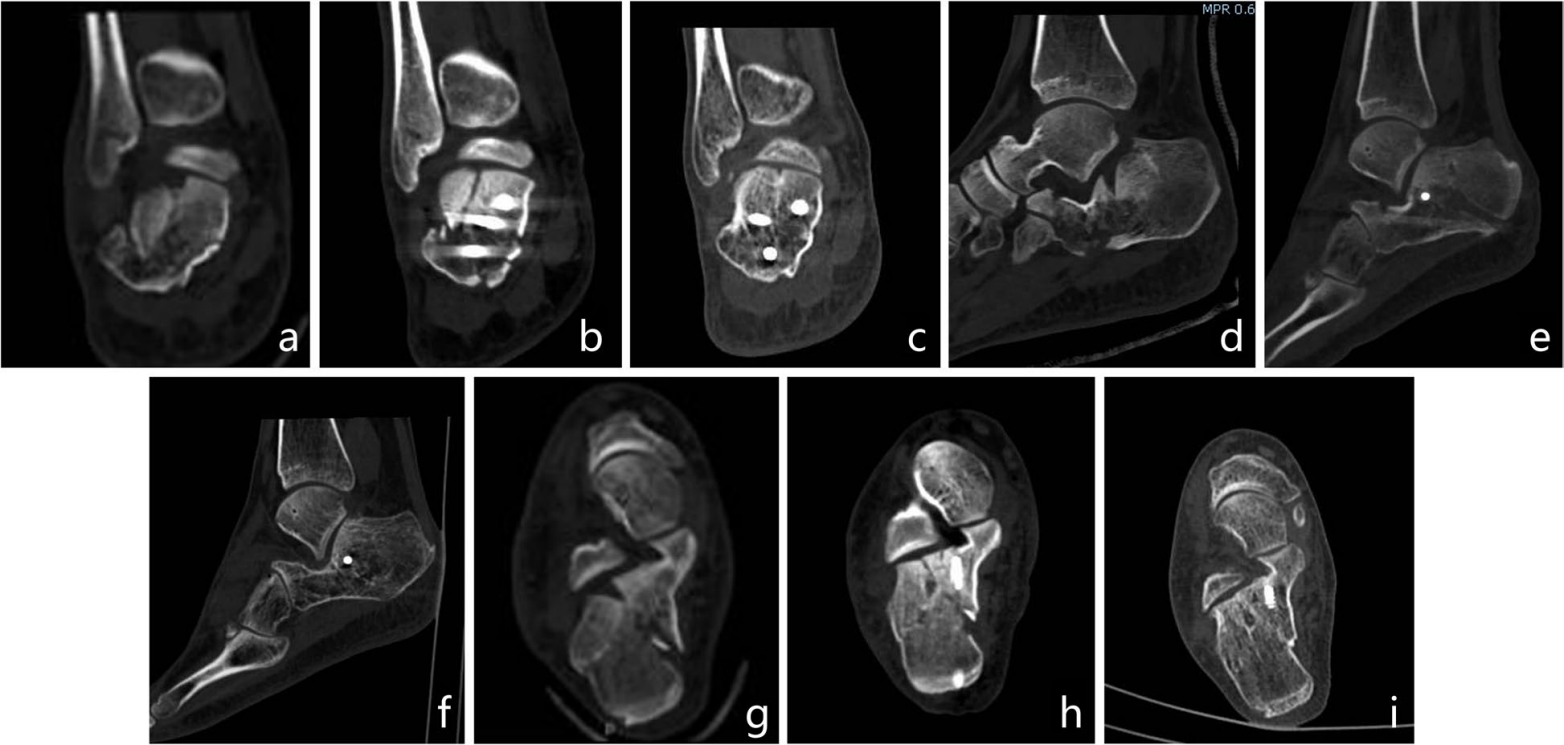
A 37-year-old male patient, Essex-Lopresti classification (joint compression type), Sanders classification (II B). Preoperative CT: coronal (**a**), sagittal (**b**) and axial (**c**) views showed significantly decreased calcaneal height and BÖhler angle. Immediate postoperative CT:coronal (**d**), sagittal (**e**) and axial (**f**) views showed calcaneal anatomical reduction,sagittal (**e**) view showed a cavity has been present in the neutral triangle after reduction.17 months postoperative CT:coronal (**g**), sagittal (**h**) and axial (**i**) views showed fracture has healed without reduction lose, sagittal (**h**) view showed the cavity has been filled with massive cancellous bone

Figure 6 shows the typical case.

## Discussion

**Fig. 7:**
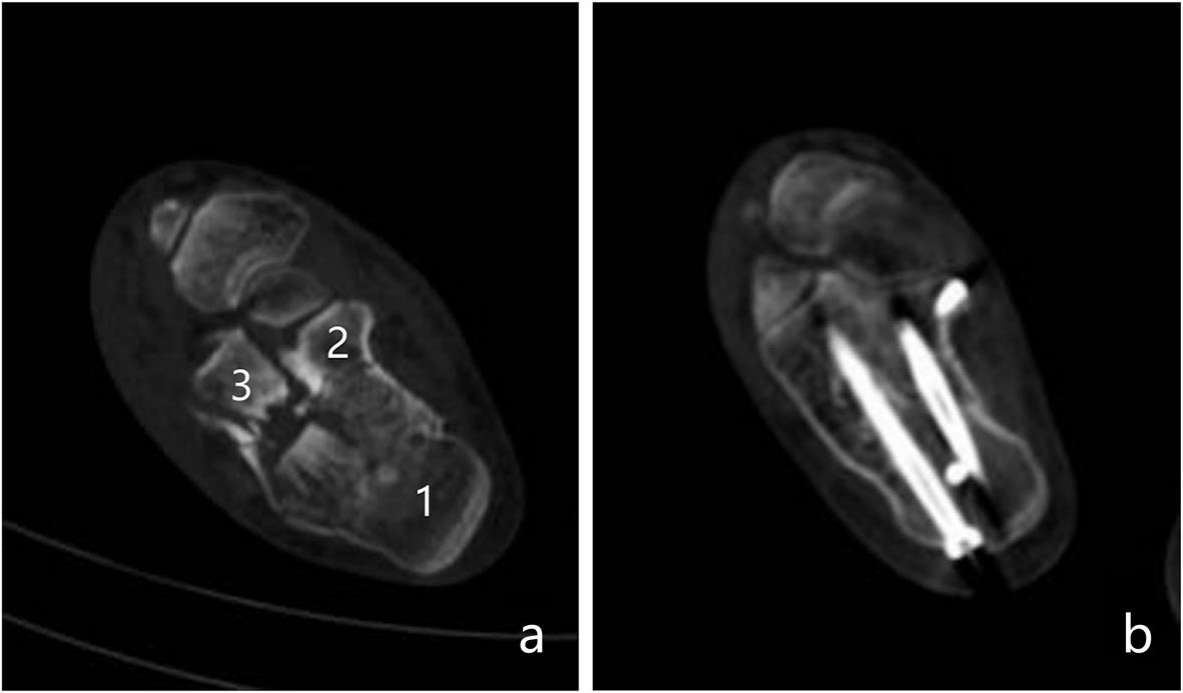
**a** The core fracture blocks:calcaneal tuberosity fracture block (1), sustentaculum tali fracture block (2), anterior calcaneal fracture block (3). **b** The core fracture blocks were integrated with reasonably distributed screws

As with any articular fracture, in calcaneal fracture, depressed articular fragments crush the weak subchondral bone underlying the posterior facet, leaving a mean central defect of 11 cc following surgical reduction [15, 26–28]. If internal fixation is not adequately stable, studies show that the presence of this bone void may predispose the calcaneus to collapse, resulting in loss of both posterior facet reduction and calcaneal height [15, 29, 30]. Therefore, some scholars advocate using bone-graft or substitute to enhance mechanical strength, whether using autogenous iliac bone, allogeneic bone, or other substitutes [20]. In this study, all DIACFs underwent percutaneous reduction and screw fixation without bone grafting. However, we did not find articular surface collapse, and the BÖhler angle and calcaneal height both well maitained during the short- and medium-term follow-up. We thought there may be the following reasons: (1) Bone grafting is needed to further stabilise the fracture only when the bone defect is serious after fracture reduction and the strength of internal fixation is insufficient [31, 32]. (2) Percutaneous screw fixation can achieve sufficient stability has been confirmed by a large number of studies [23, 33, 34]. Smerek J P [33] confirmed that there not a significant difference in the fixation strength obtained from screws and plates for Sanders type-II B calcaneal fractures. Tornetta [34] treated 46 patients (39 cases of Sanders type-IIC and 7 cases of type-IIB) with percutaneous screw fixation and believed that for Sanders type-IIC calcaneal fractures, percutaneous screw fixation has the same or even a better effect than open reduction and internal fixation. Abdelgaid [23] applied percutaneous screw fixation to treat 60 patients with DIACFs and believed that this technique is suitable for most types of calcaneal intra-articular fractures. (3) We had used the key point fixation principle of core fracture blocks to strengthen fixation. The core fracture blocks (calcaneal tuberosity fracture block, sustentaculum tali fracture block, anterior calcaneal fracture block) (Fig. 7) were integrated with reasonably distributed screws to increase the fixation strength of the screws. The first screw was used to fix the articular fracture fragment from the calcaneal colliculus rather than the lateral wall towards the sustentaculum tali. Because the calcaneal colliculus has a concentration of radial trabeculae from the calcaneal body, the bone is dense, and therefore, the fixation is stronger. The second screw points from the medial side of the calcaneal tuberosity to the sustentaculum tali along the medial wall of the calcaneus. Since the cortical bone of the sustentaculum tali is hard and forms a strong medial bearing column together with the medial wall of the calcaneus, this screw can better maintain the height of the calcaneus. The third screw points from the calcaneal tuberosity to the anterior tubercle of the calcaneum. Because the trabeculae of the anterior tubercle are richer and more rigid, fixation is more effective and can better maintain the length of the calcaneus. In addition, the screw passes through the neutral triangle of the calcaneum, located below the collapsed articular surface, and it also plays a role in supporting the articular fracture to some extent.

Other supporters of bone grafting believe that it can fill the cavity, promote fracture healing [20]. However, in our present study, all fractures healed without bone grafting. This maybe due to the following reasons: (1) Although a large cavity will be present in the neutral triangle after reduction, due to the greater healing potential in cancellous bone and the tremendous angiogenic potential of trabecular bone, which is enough to fill the void over time, fracture healing can be observed 4–8 weeks postoperatively on imaging, even without bone grafting [8, 35, 36]. (2) In the present study, we used an indirect reduction technique to reduce the articular surface fracture fragments. On the one hand, further bone loss caused by the operation can be prevented, and the volume of the cavity formed by reduction can be decreased. On the other hand, indirect reduction can avoid the destruction of blood supply in the fracture area. (3) We used a few tricks in the reduction of articular fragments about joint depression-type DIACFs. A 2.0 mm K-wire was drilled under lateral and axial fluoroscopy of the calcaneus to ensure that it was in the best position to reduce the compressed fracture fragment. Then, a hollow punch was inserted along the k-wire to reduce the compressed fracture fragment (Fig. 3). This could decrease the bone loss caused by the repeated insertion of punch.

**Fig. 8:**
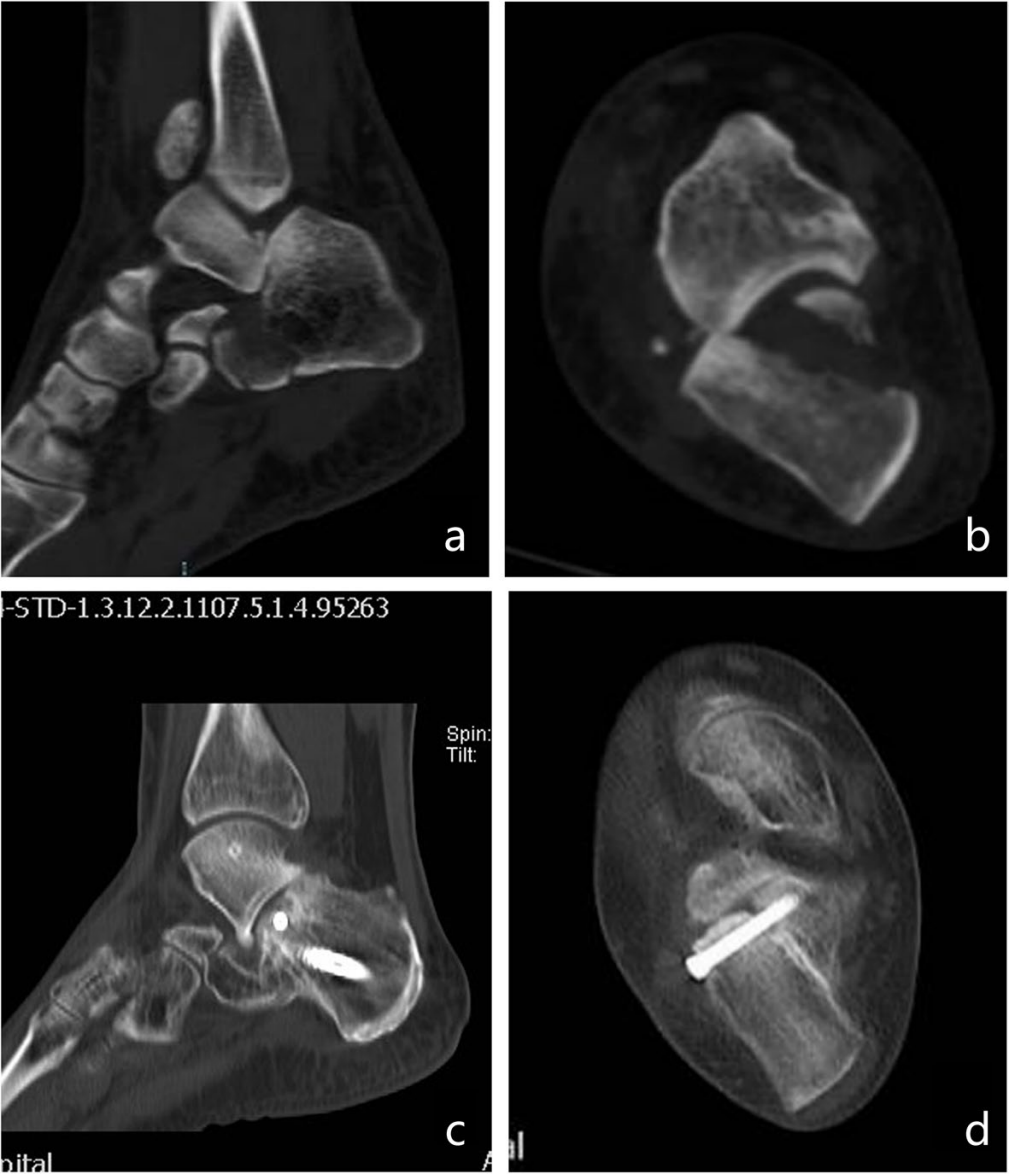
Preoperative CT. sagittal (**a**), axial (**b**) views and 1 year postoperative CT.sagittal (**c**), axial (**d**) views of a 38 years old female patient who was suffered traumatic arthritis

Incision complications are the most common complications in the surgical treatment of DIACFs, especially in ORIF with the extended lateral approach, with an incidence of 27%-33% [22]. Patients with poor blood glucose control, peripheral vascular disease, long-term smoking and heavy soft tissue injury had a higher incidence of this complication. Percutaneous minimally invasive treatment addresses this problem well, reducing the complications to 0–15% [24, 25]. In the present study, no patient experienced incision complications, which is basically consistent with literature reports. One patient had pain in the hindfoot one year postoperatively, manifesting as pain in the tarsal sinus area when walking on an uneven road surface, and oral nonsteroidal anti-inflammatory painkillers significantly improved the symptoms. Radiographically, traumatic arthritis was seen, but the articular surface was flat (a step-off of < 2 mm). The calcaneum height and BÖhler's angle showed no significant changes compared with those immediately postoperatively. A review of the history suggested that the patient had subluxation of the subtalar joint when injured, with severe damage to the calcaneal articular cartilage (Fig. 8). We believe that this may be closely related to the occurrence of traumatic arthritis in this patient.

The limitations of this study are as follows: First, it was a single-center retrospective study and the sample size was small. Second, the Sanders classification was taken as one of the inclusion criteria. Although the Sanders classification can be used as a standard to reflect the severity of DIACFs, it cannot be used as a standard to judge the size of the cavity left after the reduction of articular surface fracture fragments, and the size of the cavity is also one of the criteria for determining whether bone grafting is needed. Some scholars believe that the average cavity needed for bone grafting is about 10 cc [15]. In future studies, we need to find a way to judge the size of the cavity and what portion of the cavity requires bone grafting. Third, this study was not long enough. It is unclear whether collapse will occur with a longer follow-up period, as described by Wang and Wei [37], who found that the BÖhler angle had decreased at a mean follow-up of 40.4 months compared with the values obtained immediately postoperatively.

## Conclusions

We found that percutaneous redution and screw fixation without bone grafting in the treatment of Sanders Type-II and Type-III DIACFs provides good functional outcomes, manifested as anatomic reconstruction of the BÖhler and the height of the calcaneum. The results of the present study suggest that bone grafting is not necessary with percutaneous reduction and screw fixation in the treatment of DIACFs. However, due to the limitations of this study, the above conclusions need to be further validated by high-quality prospective controlled study with large sample and long follow-up.

## Data Availability

The datasets supporting the conclusions of this article are included within the article.

## Abbreviations

AOFAS: American Orthopaedic Foot and Ankle Society
CT: Computed Tomography
DIACFs: Displaced Intra-articular Calcaneal Fractures
MFS: Maryland Foot Score
ORIF: Open Reduction and Internal Fixation
TCDR: Tri-plane Calcaneal Distraction Reductor
VAS: Visual Analog Scale
